# Salt Loading in Canola Oil Fed SHRSP Rats Induces Endothelial Dysfunction

**DOI:** 10.1371/journal.pone.0066655

**Published:** 2013-06-07

**Authors:** Annateresa Papazzo, Xavier A. Conlan, Louise Lexis, Fadi J. Charchar, Paul A. Lewandowski

**Affiliations:** 1 School of Medicine, Deakin University, Victoria, Australia; 2 Centre for Chemistry and Biotechnology, School of Life and Environmental Sciences, Deakin University, Victoria, Australia; 3 Department of Human Biosciences, La Trobe University, Victoria, Australia; 4 School of Health Sciences, University of Ballarat, Ballarat, Australia; Wageningen University, The Netherlands

## Abstract

This study aimed to determine if 50 days of canola oil intake in the absence or presence of salt loading affects: (1) antioxidant and oxidative stress markers, (2) aortic mRNA of NADPH oxidase (NOX) subunits and superoxide dismutase (SOD) isoforms and (3) endothelial function in SHRSP rats. SHRSP rats were fed a diet containing 10 wt/wt% soybean oil or 10 wt/wt% canola oil, and given tap water or water containing 1% NaCl for 50 days. Without salt, canola oil significantly increased RBC SOD, plasma cholesterol and triglycerides, aortic p22*^phox^*, NOX2 and CuZn-SOD mRNA, and decreased RBC glutathione peroxidase activity. With salt, canola oil reduced RBC SOD and catalase activity, LDL-C, and p22*^phox^* mRNA compared with canola oil alone, whereas plasma malondialdehyde (MDA) was reduced and RBC MDA and LDL-C were higher. With salt, the canola oil group had significantly reduced endothelium-dependent vasodilating responses to ACh and contractile responses to norepinephrine compared with the canola oil group without salt and to the WKY rats. These results indicate that ingestion of canola oil increases O_2_^−^ generation, and that canola oil ingestion in combination with salt leads to endothelial dysfunction in the SHRSP model.

## Introduction

Evidence has shown that ingestion of canola oil as the sole dietary fat source (added at 10% wt/wt to standard rat chow) shortens the life span of stroke-prone spontaneously hypertensive (SHRSP) rats compared to soybean oil or perilla oil.[1]–[6] We have previously shown that canola oil ingestion reduces the lifespan of SHRSP rats compared to soybean oil following 1% NaCl loading, 85.8±1.1 and 98.3±3.4 days, respectively.[7]

The SHRSP rat is a model of human essential hypertension and stroke. [3] It is often used to model metabolic disease in humans and understanding the mechanism by which canola oil reduces life span is of significant importance. Decreased antioxidant activity and heightened oxidative stress have been implicated, since short-term ingestion (25 days) of canola oil results in a decrease in RBC superoxide dismutase (SOD) and glutathione peroxidase GPx activity, coupled to an increase in LDL-C and total cholesterol.[8] The concentration of phytosterols in canola oil has also been suggested to be a contributing factor to the shorten lifespan observed in SHRSP when consuming the oil. However, conflicting results have shown no clear correlation between the content of phytosterols in the diet and tissues and survival time observed. [9], [10] In addition, it has been common practice that SHRSP fed canola oil also receive a 1% NaCl loading to accelerate the development of hypertension. [1]–[6] A potential shortcoming of previous studies is the NaCl loading may be masking the effects of canola oil consumption alone in the SHRSP rat. A recent study showed that dietary phytosterols and phytostanols increase blood pressure in WKY rats without NaCl loading. [11]

We have previously shown that in the presence of salt, canola oil ingestion leads to an increase in malondialdehyde (MDA) and a decrease in NAPDH oxidase (NOX) and SOD aortic mRNA expression.[8] There is growing evidence that oxidative stress leads to vascular damage and plays a critical role in the pathogenesis of CVD such as hypertension, and is elevated in SHRSP rats.[12] An increase in ROS activity contributes to the impaired regulation of physiological processes within the vascular wall, which leads to structural and functional changes observed in hypertension.[13] Increased ROS activity in the vasculature can lead to a decrease in nitric oxide (NO) bioavailability and impaired endothelium-dependent vasorelaxation, which results in endothelial dysfunction-an independent predictor of CVD.[14] An excess of vascular ROS generation or reduced levels of antioxidants has been associated with the pathogenesis of endothelial dysfunction in SHRSP rats.[15] Furthermore, high salt intake has been shown to induce oxidative stress in salt sensitive hypertensive individuals [16] increase superoxide (•O_2_^−^) production in SHR[17] and lead to endothelial dysfunction in Sprague-Dawley rats. [18] With this in mind, it is hypothesized that canola oil ingestion in combination with salt leads to endothelial dysfunction and enhances oxidative stress.

The effect of canola oil ingestion for a longer period of time on oxidative stress in the absence or presence of excess dietary salt has not been examined previously in SHRSP rats. The aims of this study were to determine if 50 days of canola oil ingestion in the absence or together with excess dietary salt affects antioxidant and oxidative stress markers in circulation, mRNA expression of NOX subunits and SOD isoforms in the aorta, and endothelial function in SHRSP rats.

## Results

### Body Weight, Food Intake and Water Intake

**Figure 1:**
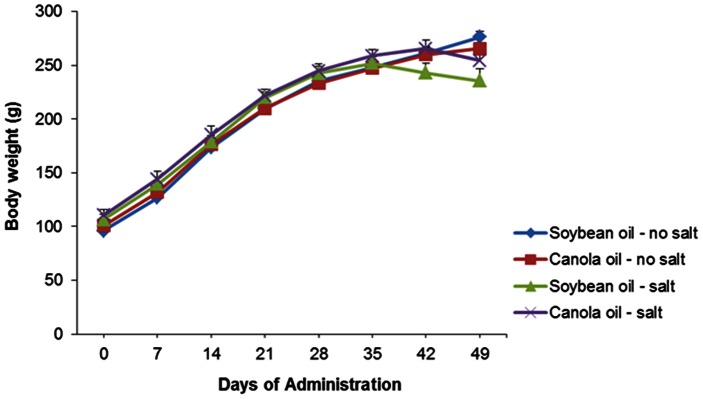
Mean body weight of SHRSP rats fed canola oil compared with soybean oil diet in the absence or presence of NaCl loading. Vaules are means ± SEM.

**Figure 2:**
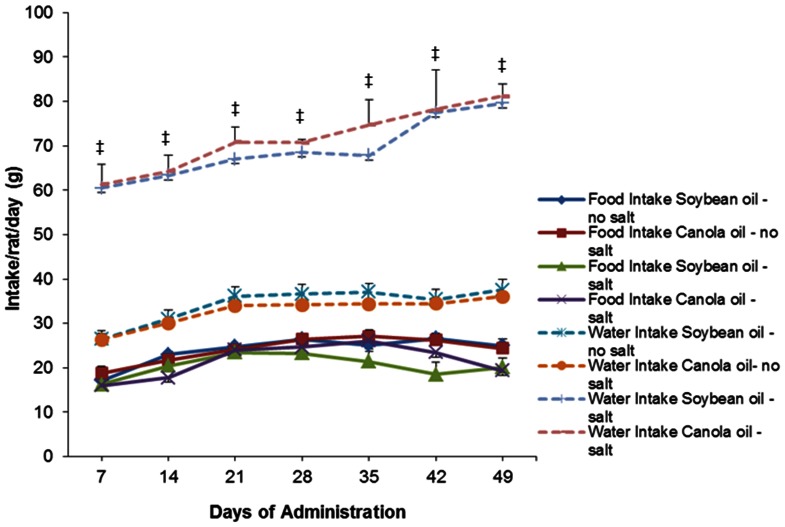
Food and water intake of SHRSP rats fed canola oil compared with soybean oil diet in the absence or presence of NaCl loading. Values are means ± SEM. ^‡^ *P*<0.05 represents a significant difference from soybean oil and canola oil no salt groups.

Body weight of the SHRSP rats increased gradually over the course of the trial in all diet groups. There were no significant differences (*P*>0.05) between soybean oil and canola oil groups (Figure 1). There were also no significant differences (*P*>0.05) in food consumption between all dietary groups (Figure 2). The water intake in soybean oil and canola oil groups with salt was significantly increased (*P*<0.05) between the soybean oil and canola oil groups without salt (Figure 2).

### Blood Pressure

**Figure 3:**
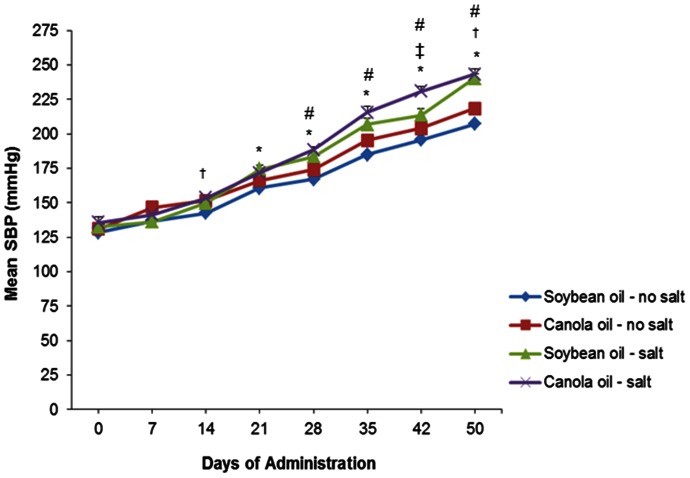
Mean systolic blood pressure of SHRSP rats fed canola oil for 50 days compared with soybean oil in the absence or presence of salt. Vaules are means ± SEM. ^†^ *P*<0.05 represents a significant difference between canola oil no salt and soybean oil no salt; ^#^ *P*<0.05 represents a significant difference between canola oil no salt and canola oil with salt; *P<0.05 represents a significant difference between soybean oil no salt and soybean oil with salt; ^‡^ *P*<0.05 represents a significant difference between canola oil with salt and soybean oil with salt.

Blood pressure increased over time in all dietary groups (Figure 3). At day 14, the blood pressure was significantly increased (*P*<0.05) in the canola oil group without salt (151.6±1.9 mmHg) compared to the soybean oil group without salt (142.6±2.1 mmHg). From day 21 to day 50, the blood pressure was significantly increased (*P*<0.05) in the soybean oil group with salt compared to the soybean oil group without salt. Also, from day 28 to day 50, the blood pressure was significantly increased (*P*<0.05) in the canola oil group with salt compared to the canola oil group without salt. At day 42, the blood pressure was significantly increased (*P*<0.05) in the canola oil group with salt compared to the soybean oil group with salt.

### Antioxidant Enzymes and Oxidative Damage

Markers of antioxidant status and oxidative damage are represented in Table 1. In the absence of salt, canola oil ingestion significantly increased (*P*<0.05) the activity of RBC SOD and significantly reduced (*P*<0.05) the activity of RBC GPx compared with soybean oil alone. In the presence of salt, RBC SOD and catalase were significantly reduced (*P*<0.05) in the canola oil group compared with the canola oil group without salt. The RBC SOD activity for each animal is shown in Figure S1. RBC GPx and catalase were significantly reduced (*P*<0.05) in the soybean oil group with salt compared with the soybean oil group without salt. Canola oil ingestion with salt significantly increased (*P*<0.05) RBC MDA compared to the soybean oil group with salt and to the non-salt groups. Plasma MDA in the canola oil group with salt was significantly lower (*P*<0.05) compared to the soybean oil group with salt. The RBC MDA concentration for each animal is shown in Supplementary Figure S2. Soybean oil intake with salt significantly increased (*P*<0.05) plasma 8-isoprostane compared to the canola oil group with salt and to the non-salt groups.

**Table 1:** Blood biochemistry in SHRSP rats fed canola oil for 50 days compared with soybean oil diets in the absence and presence of NaCl loading.

	Soybean oil no salt	Canola oilno salt	Soybean oil salt	Canola oilsalt
RBC SOD (U/gm Hb)	281±48	426±53*	297±45	290±31^#^
RBC GPx (mmol/min/gm Hb)	140±9	110±9*	74.1±12.5*	96.6±17.1
RBC Catalase (mmol/min/gm Hb)	455±54	382±35	302±27*	216±36*^, #^
RBC MDA (µM)	14.7±1.3	17.7±2.2	17.6±1.9	34.5±5.8^†, ‡^
Plasma MDA (µM)	18.8±0.6	18.8±1.6	19.6±0.6	16.1±0.8^†^
Plasma 8-isoprostane (pg/ml)	110±9	97.7±15.3	155±18^†, ‡^	87. ±10.9
Total cholesterol (mmol/L)	2.8±0.1	3.2±0.1*	4.2±0.3*	3.5±0.2^†^
LDL-C (mmol/L)	1±0.04	1.2±0.1	2.1±0.2*	1.7±0.2^‡^
HDL-C (mmol/L)	1.4±0.04	1.5±0.04	1.6±0.06*	1.6±0.06
Triglycerides (mmol/L)	1.5±0.1	2.1±0.1*	2±0.2*	2±0.1*

Values are means ± SEM. ^†^ *P*<0.05 represents a significant difference between canola oil and soybean oil with salt groups; ^#^ *P*<0.05 represents a significant difference from canola oil no salt group; ^‡^ *P*<0.05 represents a significant difference from soybean oil and canola oil no salt groups. *P<0.05 represents a significant difference from soybean oil no salt group.

### Plasma Lipids

Canola oil ingestion alone significantly increased (*P*<0.05) the concentration of total cholesterol and triglycerides compared to soybean oil alone. Total cholesterol was significantly higher (*P*<0.05) in the canola oil group with salt compared to the canola oil group without salt. LDL-C was significantly increased (*P*<0.05) in the canola oil group with salt compared to both the canola oil and soybean oil groups without salt. The concentration of triglycerides was significantly lower (*P*<0.05) in the canola oil group with salt compared to the soybean oil group with salt. In the soybean oil group in the presence of salt, the concentration of total cholesterol, LDL-C, HDL-C and triglycerides were significantly higher (*P*<0.05) compared to soybean oil alone (Table 1).

### Aortic mRNA Gene Expression

Canola oil without salt significantly increased (*P*<0.05) p22*^phox^* and NOX2 mRNA expression compared to soybean oil no salt (Table 2). Both the canola oil and soybean oil groups with salt significantly reduced (*P*<0.05) the mRNA expression of p22*^phox^* compared to the canola oil group without salt. NOX2 mRNA expression was significantly increased (*P*<0.05) in the soybean oil group with salt compared to both the soybean oil and canola oil groups without salt. CuZn-SOD mRNA expression was significantly increased in both the canola oil and soybean oil groups with salt (*P*<0.05) compared to the soybean oil group without salt. There were no changes seen in the mRNA expression of Mn-SOD and Ec-SOD between groups.

**Table 2:** Effect of 50 days of canola oil intake compared to soybean oil intake on mRNA expression in the aorta of SHRSP rats in the absence and presence of NaCl loading.

Gene	mRNA expression (Arbitrary units)			
	Soybean oil no salt	Canola oilno salt	Soybean oilsalt	Canola oilSalt
p22*^phox^*	7.6±1.4	21.8±3.5*	8.9±2^#^	11.5±4.1^#^
NOX2	1.4±0.6	12.1±5*	65±22.6^‡^	26.2±6
CuZn-SOD	22.2±5.1	39.2±7.8*	60.5±15.9*	54.9±15*
Mn-SOD	76±19.9	56.7±9.1	100±18.6	95.2±22.2
Ec-SOD	7.1±1.9	4.4±0.9	8.1±1.7	7.6±1.8

Values are mean ± SEM. ^‡^ *P*<0.05 represents a significant difference from soybean oil and canola oil no salt groups; *P<0.05 represents a significant difference from soybean oil no salt group; ^#^ *P*<0.05 represents a significant difference from canola oil no salt group.

### Vascular Function

**Figure 4:**
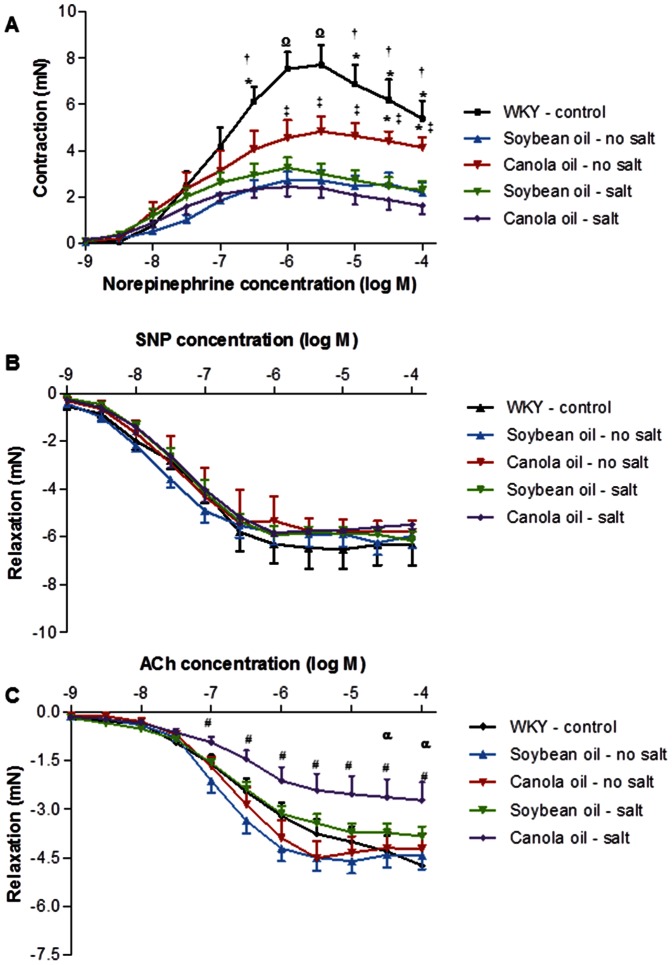
Cumulative concentration-response curves for norepinephrine (A), SNP (B) and ACh (C) in aortic rings from SHRSP and WKY rats. Values are mean ± SEM. *P<0.05 represents a significant difference from soybean oil no salt; ^†^ *P*<0.05 represents a significant different from canola oil and soybean oil with salt; ^#^ *P*<0.05 represents a significant difference from canola oil no salt; ^‡^P<0.05 represents a significant difference from canola oil with salt; ^Ω^ *P*<0.05 represents a significant difference from all dietary groups; ^α^ *P*<0.05 represents a significant difference from WKY rats.

Canola oil fed SHRSP rats with salt intake showed significantly reduced (*P*<0.05) contractile responses to norepinephrine compared to SHRSP rats fed only canola oil (Figure 4A). The contractile responses to norepinephrine were significantly greater (*P*<0.05) in the SHRSP rats fed only canola oil compared to the SHRSP rats fed only soybean oil. Contractile responses to norepinephrine were significantly greater (*P*<0.05) in the WKY rats compared to the SHRSP rats fed canola oil and soybean oil in the presence of salt and to the soybean oil only group. Relaxation responses to SNP were not significantly different between groups (*P*>0.05) (Figure 4B). The ACh dilating responses were not significantly different (*P*>0.05) between the SHRSP rats fed canola oil and soybean oil without salt (Figure 4C). Canola oil fed SHRSP rats with salt loading showed a significantly impaired (*P*<0.05) endothelium-dependent relaxation response of the thoracic aorta compared to the canola oil only group and WKY rats.

## Discussion

### Antioxidant Enzymes and Oxidative Damage

In the present study, canola oil ingestion alone increased the activity of RBC SOD and reduced RBC GPx compared with the soybean oil group. Similar results have also been found in a study by Ohara et al. 2009, in which the activities of GPx and catalase were reduced in the liver of WKY rats following canola oil ingestion.[19] Furthermore, our previous study found that 25 days of canola oil ingestion alone decreased the activities of RBC SOD and GPx.[8] The results from the present study suggest that 50 days of canola oil ingestion alone leads to decreases in RBC antioxidant levels. Here we also show that combining salt and canola oil in the diet reduces the activities of RBC SOD and catalase. This data is consistent with our lifespan study data, where we showed a decrease in RBC SOD and catalase, however, RBC GPx was also reduced at the end of life.[7] Furthermore, the results from the previous lifespan study did not find any changes in the activities of the RBC antioxidants in the soybean oil group.[7] RBCs are under constant exposure to ROS and oxidative stress due to their role in transporting oxygen and carbon dioxide, and their increased content of heme iron.[20] Therefore, the oxidative/antioxidant balance can become altered. Supporting evidence shows that there is an inverse association between reduced antioxidants and CVD.[21] In addition, previous research has shown an inverse relationship between erythrocyte GPx activity and the incidence of CVD.[22]

In the present study we found that canola oil ingestion in combination with salt leads to an increase in RBC MDA compared to all the other dietary groups. However, our previous study showed that canola oil in combination with salt lead to a decrease in plasma MDA at the end of their lifespan.[7] Further to this, short-term intake (25 days) of canola oil in combination with salt has been shown to increase both RBC and plasma MDA compared to the canola oil alone.[8] Taken together, these results indicate that canola oil in combination with salt increases RBC MDA, a marker of lipid peroxidation. Salt intake may be a contributing factor to the changes, as there were no changes observed in the markers of oxidative stress in the diets without salt intake. A previous study by Kitiyakara et al (2003) observed an increase in urine MDA concentration in Sprague-Dawley rats as a result of salt intake.[17] They found a difference between low salt (0.03%) and normal salt (0.3%) groups, and between low salt and high salt (6%) groups. In addition, they also found an increase in urine 8-isoprostane levels as a result of salt intake.[17]

NOX derived O_2_^−^ is enhanced in hypertension, and this is linked with the increased expression of NOX subunits (NOX2, p22*^phox^*, p47*^phox^*).[16], [17] In the present study, canola oil intake alone increased p22*^phox^* and NOX2 expression, whilst canola oil in combination with salt intake reduced p22*^phox^* mRNA expression compared to the canola oil group without salt. This data suggests that O_2_^−^ generated from NOX is elevated in the presence of canola oil alone. In addition, our previous work has shown that short term intake of canola oil in combination with salt reduced p22^phox^ mRNA expression compared to the canola oil group without salt, which is consistent with the results from the present study. A study by Kitiyakara et al. (2003) found that salt intake in Sprague-Dawley rats lead to an increase in O_2_^−^ production.[17] This was accompanied by an increase in renal activity and mRNA expression of NOX2 and p47*^phox^*, and a decrease in CuZn-SOD and Mn-SOD mRNA expression.[17] However, the results from the present study show that canola oil ingestion without salt, and both the soybean oil and canola oil groups with salt increased the mRNA expression of CuZn-SOD compared to soybean oil alone. The increase in the mRNA expression of CuZn-SOD along with an increase in RBC SOD in the canola oil group without salt may be an adaptive response to an increase in O_2_^−^ generation. Previously we demonstrated that 25 days of canola oil ingestion with salt reduced the mRNA expression of CuZn-SOD, Mn-SOD and Ec-SOD,[8] which is not evident in the present study. There were no changes found in Mn-SOD and Ec-SOD in the present study. Furthermore, in the present study, ROS generation in the canola oil group in the presence of salt may be coming from other sources within the vasculature, such as xanthine oxidase, uncoupled nitric oxide synthase, lipoxygenase and the mitochondrial respiratory chain.[23], [24] In addition, it would have been ideal to examine the protein levels of the genes of interest. However, there was insufficient protein to carry out the western blot analysis.

### Vascular Function

The results from the present study suggest that contractile responses to norepinephrine were reduced in the canola oil group with salt compared to the canola oil only group, indicating smooth muscle cell contractile dysfunction. In addition, in the present study the contractile responses to norepinephrine were greater in the SHRSP rats fed only canola oil compared to the soybean oil only group. Also, the WKY rats had greater contractile responses compared to the soybean oil only group and to both the canola oil and soybean oil groups with salt. These results show that SHRSP rats have reduced smooth muscle contractile function; however, the intake of canola oil alone may result in reducing contractile dysfunction. Furthermore, a study by Natio et al. (2000) found that in SHRSP rats, in which salt loading was not used, the contractile responses in isolated mesenteric vascular bed to norepinephrine and to other agonists such as angiotensin II, arachidonic acid, ATP, endothelin-1 or serotonin were not different between the canola oil and soybean oil group after 4 weeks of feeding.[25] The results from the present study also show that canola oil ingestion in combination with salt leads to a decrease in the endothelium-dependent vasodilating response to ACh compared to canola oil ingestion alone. The results suggest that salt intake in combination with canola oil may result in endothelial dysfunction. A study by Naito et al (2000) found that in SHR and WKY rats the endothelium-dependent and endothelium-independent vasodilating responses to ACh and SNP, respectively, were not different between the canola oil and soybean oil groups in each strain following salt loading.[26] There were no changes found in the vasodilating responses to ACh and SNP between the canola oil and soybean oil groups with salt. However, the vasodilating responses to ACh were reduced in the canola oil group with salt compared with the WKY rats. In addition, studies have shown an association between high salt intake and endothelial dysfunction.[27] A study has found that long-term high salt intake caused a reduction in endothelium-dependent vasorelaxation in salt-sensitive rats.[28] Evidence has shown that in Sprague-Dawley rats, high salt intake leads to endothelial dysfunction by reducing the concentration of NO and eNOS activity.[18] It is well established that ROS interacts with endothelial NO, thus decreasing the vasodilating response of blood vessels. However, p22*^phox^* mRNA expression was reduced in the canola oil group with salt, suggesting a decrease in NOX derived O2^−^ generation. The interaction between salt intake and canola oil is not clearly understood, however, it seems that canola oil in combination with salt leads to reduced smooth muscle contractile function and impaired endothelium-dependent vasorelaxation.

### Plasma Lipids

Previous studies have reported an increase in plasma lipids due to canola oil ingestion.[19], [25], [29], [30] Canola oil is considered to provide protective cardiovascular effects due to its favourable fatty acid composition.[6] However, in the present study, canola oil ingestion alone increased the concentration of total cholesterol and triglycerides compared with soybean oil alone. These results are consistent with the results from our previous study, which showed that short term ingestion of canola oil alone increased total cholesterol as well as LDL-C.[8] Evidence has shown that dietary salt restriction from 10 g to 2 g per day for 5 days in patients with essential hypertension significantly increased total cholesterol and LDL.[31] Furthermore, there is a positive association between increased concentrations of plasma total cholesterol, triglycerides and LDL-C and the development of CVD such as atherosclerosis.[32] In addition, the present study has also shown that canola oil ingestion in the presence of salt increased total cholesterol and LDL-C, whereas the concentration of triglycerides was reduced. Our previous life span study showed that canola oil ingestion following salt loading reduced total cholesterol and LDL-C compared with soybean oil.[7] In addition, the results from the present study also show that soybean oil in the presence of salt increased total cholesterol, LDL-C, HDL-C and triglycerides compared to soybean oil alone. In the present study, salt intake had an interacting effect on the plasma lipids, as the increase in the total cholesterol and LDL-C is common in both the canola oil and soybean oil groups with salt loading. The mechanism to explain the salt interaction and the increase in plasma lipids is unknown, and warrants further investigation.

### Blood Pressure

The association between salt intake and hypertension is well known,[33] which is evident in the present the study. The results of the present study show an increase in systolic blood pressure in both the canola oil and soybean oil groups with salt compared to the dietary groups without salt at the end of the feeding trial. Evidence indicates that canola oil intake has an effect on blood pressure in the SHRSP rat and its related strains.[3], [25], [26] However, the blood pressure in the canola oil group was not consistently different from soybean oil. Taken together these results suggest that canola oil intake in the presence or absence of salt does not affect blood pressure. This is also supported by other studies, which have shown no significant changes in blood pressure between the canola oil and soybean oil groups in SHRSP rats.[1], [5]

### Conclusion

In conclusion, canola oil intake alone increases aortic p22*^phox^* and NOX2 mRNA expression, suggesting an increase in O_2_^−^ generation. The interaction between salt intake and canola oil is not clearly understood, but it appears that canola oil ingestion in combination with salt leads to endothelial dysfunction. ROS generation in the canola oil group in the presence of salt may be coming from other sources within the vasculature, which warrants further investigation.

## Materials and Methods

### Animal Husbandry and Study Design

#### Fifty day treatment experiment

Approval for this project was granted by the Deakin University Animal Welfare Committee (Approval no. A67/09). Forty male SHRSP rats (Deakin University, Australia) aged 28 days were randomly assigned to either group 1 (n = 20) or group 2 (n = 20). Male rats were used to replicate previous experiments by our group and previous studies carried out that fed canola oil to SHRSP. [1]–[6] Within each group the rats were randomly assigned to a control and treatment group and acclimatized for one week. During acclimatization they were given a standard pellet diet (Specialty Feeds, Western Australia) and water ad libitum. The groups were then randomly subdivided with half the animals in each group fed, a defatted control diet containing 10 wt/wt% soybean oil or a defatted treatment diet containing 10 wt/wt% canola oil (Speciality Feeds) for 50 days. Fifty days was also chosen based on one of our previous studies that demonstrated the mean lifespan of the canola oil group was 85±1.1 days, when consumption of the canola oil diet commenced at 28 days of age. Soybean oil was chosen as the control oil, as it has been used in previous lifespan studies, and has been shown to have no life shortening effect in the SHRSP rat. [1]–[6] The fatty acid composition of the diets was the same as used in previous experiments by the authors. [7] Group 1 was given water containing 1% NaCl and group 2 was given tap water throughout the trial. The group without NaCl in the drinking water was included to rule out any interfering factor the salt loading may have when analysing the tissue. The animals were maintained on a 12 hr light/dark photo-period with a room temperature of 21±2°C. Animal body weights, food intake and water consumption were determined once a week, while the health of the animals was monitored daily. At the end of the 50 days the rats were anaesthetised via intra-peritoneal injection with lethabarb (50 mg/kg), and blood was collected for analysis. The aorta was then removed, washed in saline solution and snap frozen in liquid nitrogen.

#### Vascular function experiment

The dietary regime for the vascular function experiment was the same as described above for the 50 day treatment study. In brief, 40 male SHRSP rats (Deakin University, Australia) aged 28 days were randomly assigned to either group 1 (n = 20) or group 2 (n = 20) and received a standard pellet diet (Specialty Feeds, Western Australia) and water ad libitum for one week. After the acclimatization period, the SHRSP rats were fed the following diets respectively, a defatted control diet containing 10 wt/wt% soybean oil or a defatted treatment diet containing 10 wt/wt% canola oil (Speciality Feeds, Western Australia) with free access for 50 days. Group 1 was given water containing 1% NaCl and group 2 was given tap water throughout the trial. In addition a group of normotensive Wistar Kyoto (WKY) rats were fed the 10 wt/wt% soybean oil control diet for 50 days with tap water. The WKY rats were used as a normotensive control to assess endothelial function. The animals were maintained on a 12 hr light/dark photo-period with a room temperature of 21±2°C. The health of the animals was monitored daily. At the end of the 50 days the rats were anaesthetised via intra-peritoneal injection with sodium pentobarbitone (50 mg/kg). The aorta was removed and used for the isolated aortic rings experiment.

### Measurement of Blood Pressure

Blood pressure was measured weekly over the course of the both experiments using a tail cuff sphygmomanometer following the manufacturer's instructions (Biopac Systems, USA).

### Blood Collection and Processing

After the animals were anaesthetised, blood was collected via cardiac puncture into EDTA coated tubes. Immediately after blood collection, samples were centrifuged at 600 xg for 10 minutes at 4°C. The plasma was then removed and stored at −80°C until analysis of plasma lipids: triglycerides, total cholesterol, high density lipoprotein cholesterol (HDL-C) and low density lipoprotein cholesterol (LDL-C), and MDA. RBCs were then washed 3 times by adding an equal volume of 0.9% (w/v) NaCl, mixed carefully and centrifuged at 4°C at 600 xg for 10 minutes. The supernatant was removed and discarded. An equal volume of cold distilled water and RBCs were mixed well to lyse the cells. The hemolysate was stored at −80°C for subsequent analysis of antioxidant enzymes: SOD, catalase and GPx, and MDA.

### Red Blood Cell Antioxidant Enzymes

SOD activity was determined using a commercially available kit (Cayman Chemical Company, USA) following the manufacturer's instructions. This assay utilizes xanthine oxidase and hypoxanthine to generate superoxide radicals that are detected by tetrazolium salt with absorbance read at 540 nm using a microplate analyser (Fusion-Alpha HT, PerkinElmer, USA). One unit of SOD is defined as the amount of enzyme required to inhibit the distmutation of the superoxide radical by 50%.

Catalase activity was determined using a commercially available kit (Cayman Chemical Company, USA) following manufacturer's instructions. This method is based on the reaction of methanol with the enzyme in the presence of an optimal concentration of hydrogen peroxide. The absorbance was read at 540 nm using a microplate analyser (Fusion-Alpha HT, PerkinElmer, USA).

GPx activity was determined using a commercially available kit (Cayman Chemical Company, USA) following manufacturer's instructions. This assay is based on the oxidation of NADPH following the reduction of hydroperoxide. A decrease in absorbance at 340 nm results from oxidation of NADPH to NADP+ and the rate of this decrease is proportional to the GPx activity in the sample. The absorbance was read at 340 nm using a microplate analyser (Fusion-Alpha HT, PerkinElmer, USA) once every minute for 10 minutes.

All RBC enzyme activities were normalised to haemoglobin concentration, which was determined by adding 20 µl of 200/1 hemolysate and 480 µl of Drabkin's reagent. The sample was left to stand at room temperature for 5 minutes and the absorbance read at 540 nm using a spectrophotometer (Biochrom, UK).

### Lipid Peroxidation Analysis

MDA in plasma and RBCs was determined via high performance liquid chromatography (HPLC) according to the method of Sim et al. (2003) [34] and was performed as reported previously. [8]

Total 8-isoprostane concentrations were analysed in plasma using an enzyme immunoassay (EIA) kit (Caymen Chemical Company, USA) following manufactures instructions. The sample preparations were performed as previously described^8^. This assay is based on the competition between 8-isoprostane and an 8-isoprostane acetycholinesterase (AChE) conjugate for a limited number of 8-isoprostane -specific rabbit anti-serum binding sites. Values were expressed as pg/ml of plasma.

### Plasma Lipids Analysis

Plasma triglycerides, total cholesterol and high-density lipoprotein cholesterol (HDL-C) were determined using commercially available kits (Thermo Electron Corporation) in a 96 well plate format (Fusion-Alpha HT, PerkinElmer), following manufactures instructions.

Low-density lipoprotein cholesterol (LDL-C) was determined using the Friedewald equation: LDL cholesterol  =  Total cholesterol – HDL cholesterol – (triglycerides/5).[35]

### Reverse Transcription-Real-Time PCR Measurement of mRNA

Total RNA was isolated from the aorta using TRI reagent (Molecular Research Centre, USA) following the manufactures instructions. The aortic mRNA expression of NOX2, p22*^phox^*, CuZn-SOD, MnSOD and Ec-SOD were determined as previously reported.^8^ The primer sequences were all obtained from previous published journal articles [36]–[38] and ordered through Geneworks (Table 3).

**Table 3:** Real-time PCR primer sequences for genes of interest.

Gene	Forward primer (5′-3′)	Reverse primer (5′-3′)
NOX 2	TCAAGTGTCCCCAGGTATCC	CTTCACTGGCTGTACCAAAGG
p22*^phox^*	GCTCATCTGTCTGCTGGAGTA	ACGACCTCATCTGTCACTGGA
CuZn-SOD	TGTGTCCATTGAAGATCGTGTGA	TCTTGTTTCTCGTGGACCACC
Mn-SOD	TTAACGCGCAGATCATGCA	CCTCGGTGACGTTCAGATTGT
Ec-SOD	GGCCCAGCTCCAGACTTGA	CTCAGGTCCCCGAACTCATG

The primers were all obtained from previous published sequences and were ordered through Geneworks (Australia): NOX2,[38] p22^phox^,[36] CuZn-SOD, MnSOD and Ec-SOD.[37].

### Isolated Aortic Rings

Aortic rings were suspended in an organ bath chamber and bathed in Tydroyde solution bubbled with carbogen, and performed as previously described.^21^ Cumulative concentration-response curves were performed for norepinephrine, acetylcholine (Ach) and sodium nitroprusside (SNP). Force of contraction in the aorta was measured isometrically with force transducers (FT03C, Grass, USA) connected via amplifiers to a computer via a LabChart system (AD Instruments, Australia).

### Statistical Analysis

Statistical analysis was performed using the SPSS statistical package (version 17.0, SPSS Inc.) for repeated measures ANOVA and one-way ANOVA. The results are represented as mean ± SEM. Comparisons between groups for animal body weight, food intake and water intake data were analysed using repeated measures ANOVA with Bonferroni corrections. Significance was established at the 95% confidence level (*P*<0.05).

## Supporting Information

Figure S1 **RBC SOD activity data for individual animals.**
(TIF)

Figure S2 **RBC MDA concentration data for individual animals.**
(TIF)
