# Supplementary figures and images for: Salt Loading in Canola Oil Fed SHRSP Rats Induces Endothelial Dysfunction
Source: PLoS One. 2013 Jun 7;8(6):e66655. doi: 10.1371/journal.pone.0066655 (PMC3676377; doi:10.1371/journal.pone.0066655)

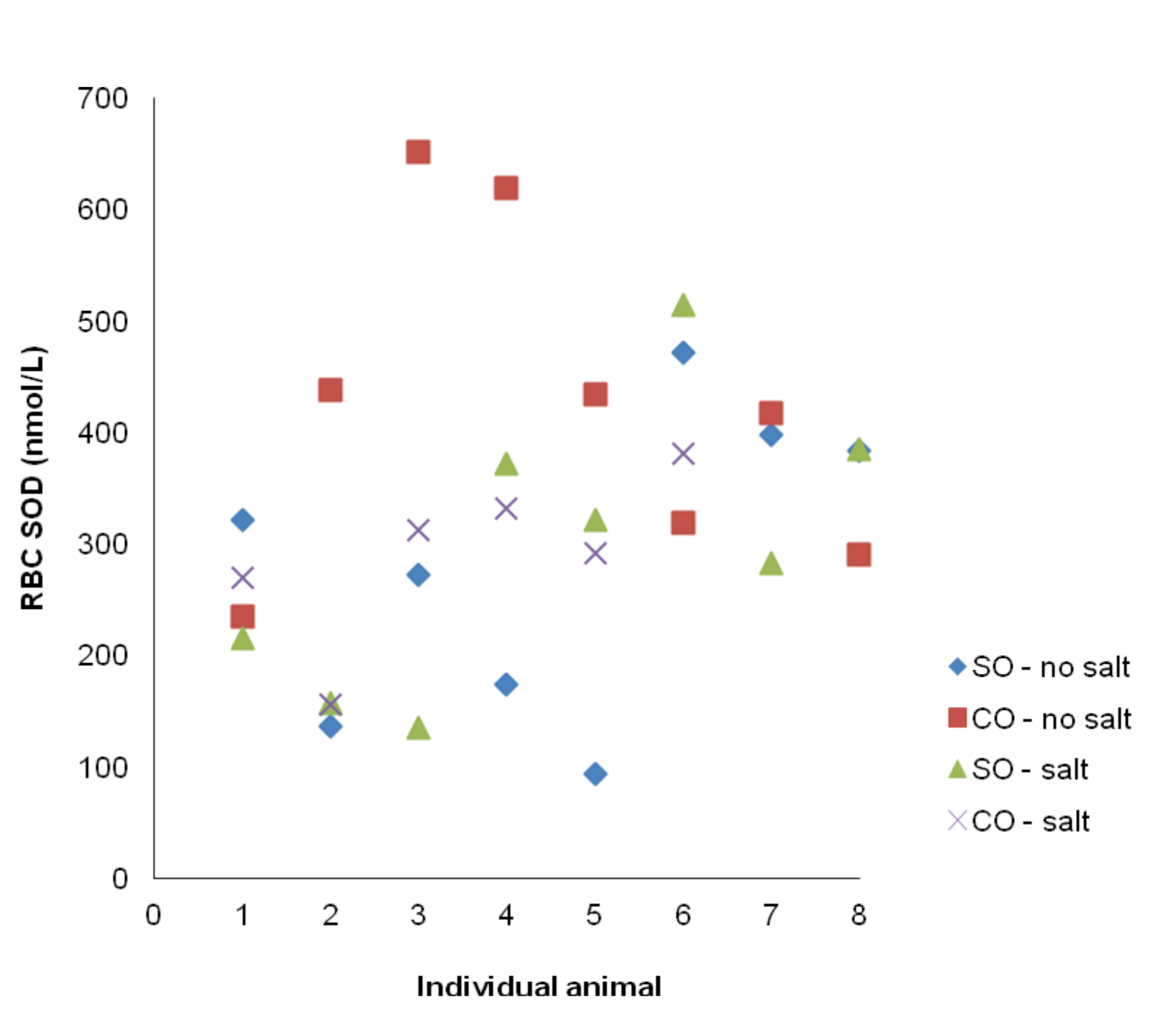

Supplement: Figure S1 — RBC SOD activity data for individual animals. (TIF) [file pone.0066655.s001.tif]

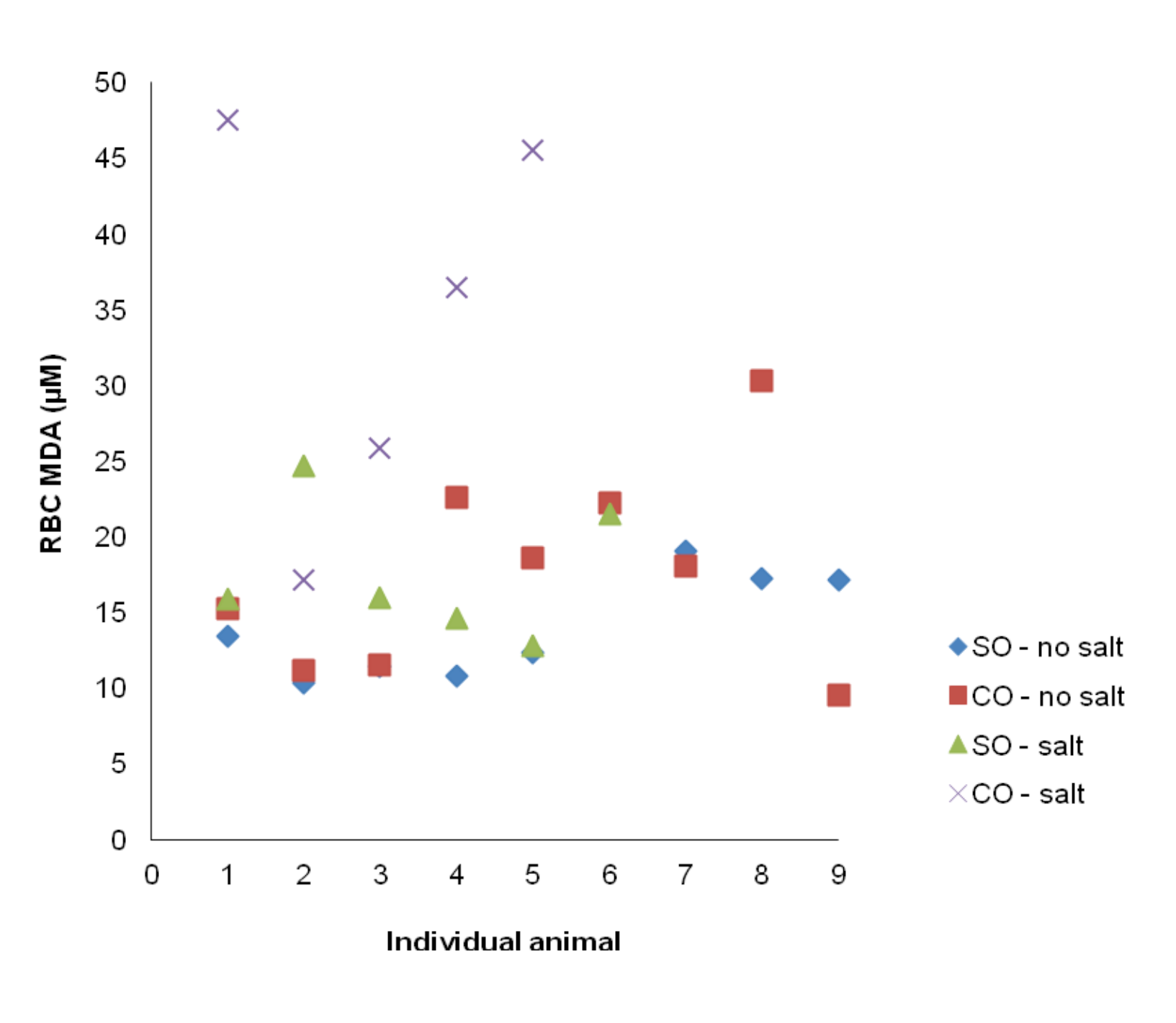

Supplement: Figure S2 — RBC MDA concentration data for individual animals. (TIF) [file pone.0066655.s002.tif]
